# Engaging older adults with a migration background to explore the usage of digital technologies in coping with dementia

**DOI:** 10.3389/fpubh.2023.1125834

**Published:** 2023-04-14

**Authors:** Catharina M. van Leersum, Kornelia E. Konrad, Egbert Siebrand, Zohrah B. Malik, Marjolein E. M. den Ouden, Marloes Bults

**Affiliations:** ^1^Science, Technology, and Policy Studies, Faculty of Behavioural, Management, and Social Sciences, University of Twente, Enschede, Netherlands; ^2^Ethics and Technology Research Group, Saxion University of Applied Science, Deventer, Netherlands; ^3^Technology, Health and Care Research Group, Saxion University of Applied Science, Enschede, Netherlands

**Keywords:** older adults, digital technology, technologies for care, citizen science, dementia, migrants, independent living aid products

## Abstract

**Background:**

Coping with dementia can imply particular challenges for people with a migration background due to diversity in their life course, personal characteristics, and living environment. Some of the services available for people with dementia include digital technologies for care, providing health services, and maintaining or increasing participation, independence, and safety. This study aimed to explore the role of digital technology in coping with dementia in the lives of older adults with a migration background, and the possibilities to engage and collaborate with older adults.

**Methods:**

This study combined a qualitative interview-based approach with citizen science principles in the design and execution of a project studying the use of Anne4Care.

**Results and discussion:**

Participants valued that technology should provide health benefits and fit into aspects of their daily lives. Anne4Care was considered helpful in staying independent and connecting to loved ones in their country of birth. The participants needed to learn new competencies to work with the device, and not all had the material prerequisites, such as an internet connection. Still, this learning process was considered purposeful in their life, and the virtual assistant could be integrated into care and daily practices. The involvement of the older adults with dementia as co-researchers made them feel valuable and as equal partners during this research. An important prerequisite for the involvement of older adults with a migration background was existing relations with carers and care organizations.

**Conclusion:**

Digital care technologies to cope with dementia can become a valuable part of care practices in the lives of older adults with a migration background. Involving older adults in the development of technology, acknowledging their expertise and needs, and working together in short iterations to adapt the technology for their specific needs and situations were experienced as valuable by the researchers, older adults, and care professionals.

## 1. Introduction

Dementia is a global problem introducing a major social and emotional burden for people with dementia, their relatives, and health authorities (1). Coping with dementia tends to imply particular challenges for people with a migration background due to diversity in their life course, personal characteristics, dietary patterns, physical activity, and living environment (2). Kenning et al. (1) found in a meta-synthesis of qualitative studies on accessing dementia care by ethnic minorities that these older adults often lacked knowledge about their disease and symptoms, and care services often lacked cultural awareness and diversity. Other issues are less familiarity with available care services and lower health literacy—someone’s ability to find, understand, and use the information to promote and maintain good health (1, 3). This is partly due to language barriers and a lack of culturally sensitive care. The language barrier often goes along with deterioration, cognitive decline, and lower levels of health literacy (2).

**Figure 1:**
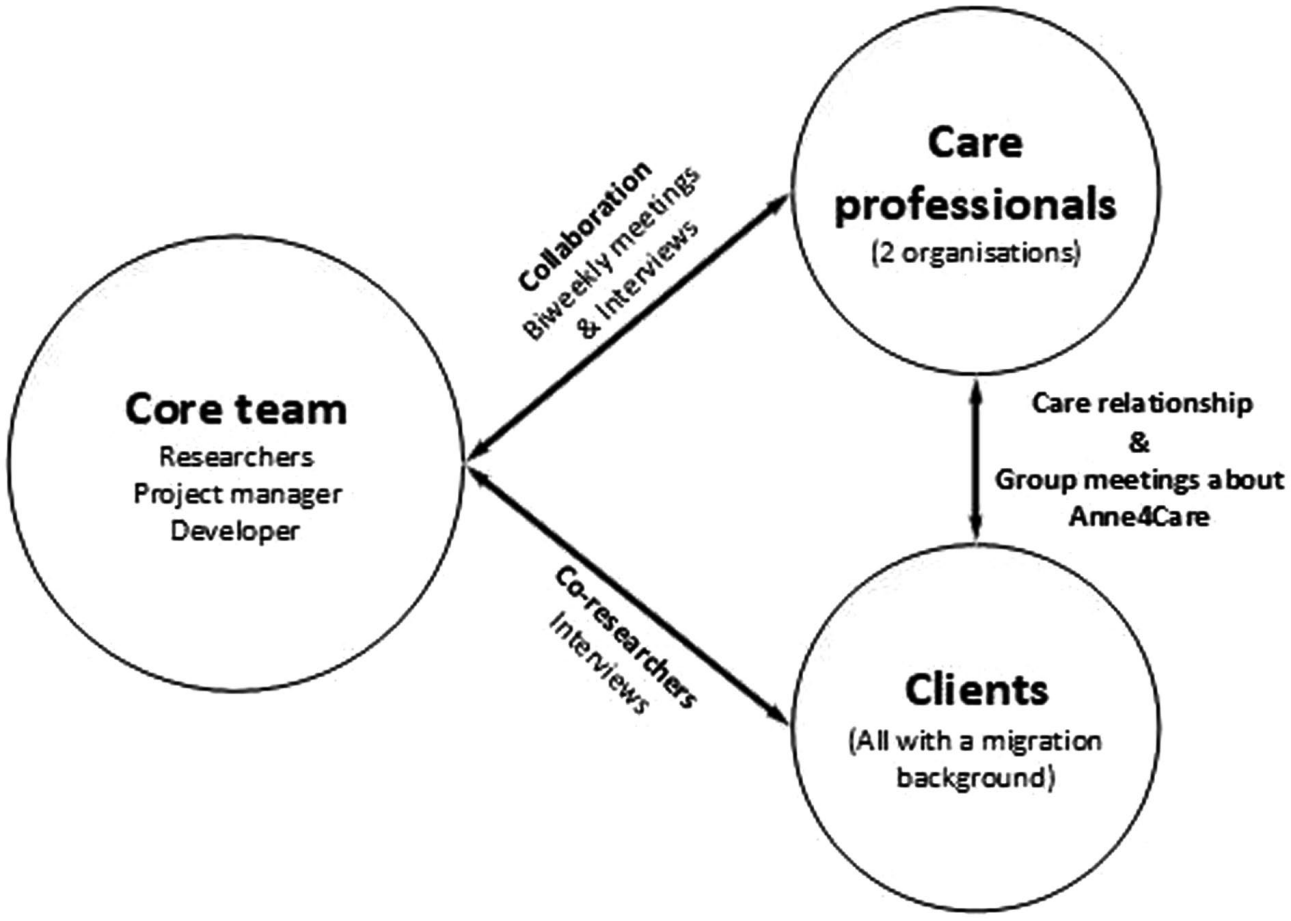
Overview of stakeholder groups and interactions.

Caring for people with dementia at home is a good option, but the need to improve access to dementia services for people with a migration background is increasing (4). With this comes a need to provide culturally sensitive care. Culturally sensitive care was described by immigrant South Asian residents in United Kingdom nursing homes as “care that respects individuality, creates mutual understanding, caters for spiritual need, and maintains dignity” (5). In a scoping review by Fang et al. (6), culturally and spiritually sensitive care was found crucial to support individuals coping with aging. They define a cultural group in terms of shared stories, beliefs, values, myths, and practices shaped by history and geography, and a spiritual group in terms of religious, spiritual, and faith-based beliefs and practices. One way to achieve culturally and spiritually sensitive care is education and skill development of care professionals with regard to diverse cultural and spiritual groups (7). Furthermore, a Dutch study showed the importance of ethnic community health workers when providing culturally sensitive care (8). Health workers from the same ethnic community share the language but, above all, are trusted and respected by the community. They act as key figures and providers of services, tackling some of the barriers migrants experience to accessing healthcare (8) (Figure 1).

Overall, with the current diverse population of people with dementia and a migration background in the Netherlands, there is a need to broaden and adapt services to meet diverse needs. Currently, more and more digital technologies for care are being introduced to older adults aimed at facilitating common tasks and activities. However, how these are received by older adults with a migration background is hardly known. The remainder of the article starts by describing the range of available digital technologies for older adults with dementia and reviewing findings on their use by older adults. Then, we introduced Anne4Care as an example technology and introduce our use of a social practices approach and citizen science as a particular approach to engage with older adults. After this introduction, we explain our methods in more detail, then the qualitative findings are presented, leading to the discussion and conclusion.

### 1.1. Digital technologies for older adults with dementia

Part of the services available for people with dementia includes digital technologies that are aimed at providing services to maintain or increase participation, activity, the feeling of independence, and safety (9). The number of available digital technologies for care is increasing rapidly and varies in form and functionality, such as eating aids, digital locks, smart home sensors, shower robots, safety bracelets, virtual doctor visits, or incontinence detection sensor technology (9, 10). Digital technologies assist older adults by providing instructions in daily or health-related tasks and life, monitoring health behavior, or providing companionship (11). These technologies have been presented as an essential means to tackle the increased demand for healthcare (10, 12), and digitalization could be one of the approaches to alleviate the challenges of the aging population and improve quality of life (13). However, older adults with a migration background tend to access and use new technologies later compared to native older adults (14, 15). One of the reasons is a low proficiency in the language in the country of residence (16). Overall, little is known about the use of digital technologies by older adults with a migration background (17, 18).

With regard to people with dementia in general, several experiments engaged people with early-stage dementia in research or development of technologies. Zamir et al. (19) showed that the use of video-calling in care environments could reduce feelings of loneliness when the family is unable to visit regularly. Technologies with virtual agents can strengthen the self-management of people with dementia and their self-care, participation, and independence (20). Stara et al. (21) explored the use of Personal Virtual Assistant (PVA) Anne4Care (see Box 1). They concluded that the use of these screen-based applications allows for natural interactions between humans and computers. Other studies have shown that exercises could be provided with the use of animated conversational agents (22), virtual agents give the user a sense of companionship (23), and people with dementia seem to naturally engage with these virtual agents (24). However, people with a migrant background make less use of the Internet or technology for health purposes and were not included as key stakeholders in development or research processes (16, 18).

**BOX 1 Anne4Care.**

Anne4Care is a VPA that includes video-calling, a personal agenda, medication reminders, reading the news, and games. It is a technological device created to support users with cognitive impairments in many aspects of their daily life. Hence, it enables them to live more actively and independently and lowers the burden on formal and informal caregivers (21). The device Anne4Care is available in different languages, such as Dutch and English. Currently, the company is developing a Turkish version of the same device and saw the embedding of Anne4Care in homes of older adults with a migration background as an opportunity to test and improve the latest version.

The COVID-19 pandemic caused more loneliness among older adults. Caregivers who were involved in this project searched for creative solutions to decrease feelings of loneliness among their clients and raised some ideas, such as introducing video-call technologies in the homes. Especially for these older adults, who were not able to visit family in their birth countries, easy access to video-call technologies appeared to be a good solution. People could benefit by using video calls to strengthen social relationships, overcome difficulties with the local language, and foster independence (18).

### 1.2. Approach: Social practices and citizen science

In our study, we aimed at an approach that allowed us to closely involve users in our research and to be sensitive to their particular experiences, lifeworlds, and needs (2, 18). In line with Shaw et al. (25), we chose a social practice orientation and combined it with the application of citizen science principles.

Wider use of technologies, such as Anne4Care, often proved more difficult than anticipated (25). To be used productively, technologies need to be embedded within the daily practices of their users and their wider socio-technical networks, accounting for situated knowledge, personal habits, and collective routines (25). Even if the technology is already in use by one stakeholder group, practices and socio-technical networks might be different for another stakeholder group. In this research, it was important to adapt the technology to the personal needs of older adults. It needs to fit with their daily practices but might influence these practices and those of others related to older adults as partners and carers as well. Social Practice theory argues that new technologies may change current practices, with practices being constituted by material elements, such as objects, tools, technologies, and infrastructures, by competencies, such as knowledge, practical know-how, and embodied skills, and by meanings, such as cultural conventions, expectations, and socially shared meanings (26, 27). All these elements are essential for a practice to “work” and to become part of people’s daily lives and social networks.

Citizen science, or the use of scientific principles and methods by non-professional scientists, may be a powerful method to improve public participation in research as well as to improve the implementation of technologies (28). Citizen science principles require close collaboration with the intended users, involving them actively in the capacity of co-researchers rather than mere research subjects. This close collaboration of researchers with co-researchers is meant to enable an alignment of the technology with the needs of the users and their practices (25, 29). Close involvement throughout the research process allows citizens to influence and improve both the design and execution of research and to prevent important aspects from being neglected or omitted, for example, aspects that are related to having a migration background and that researchers may not be aware of.

In some development processes of video-call interventions, older adults have been involved. However, in most development processes, younger adults who are not retired, do not receive care, show no signs of dementia, and have a certain level of technology literacy are usually included (30). As Fischer et al. (31) argue, the full potential of including older adults in the development of technologies is still to be exploited. This may be one of the reasons why innovative technologies struggle to meet expectations and get the attention of the aging population (31).

We did not come across any studies investigating the use of technology or involving older adults with a migration background in the development processes of digital technologies for care. Most studies include the majority population. Therefore, it remains unclear how these older adults experience being involved in the development of technologies (18). This study aimed to explore (1) the role of digital care technology in the lives of older adults with a migration background in an early stage of dementia and (2) ways to engage and collaborate with these older adults in the development of technology. Against this aim, Anne4Care served as a case example and we drew on citizen science principles to assess the perceived value of the application for the users and study the use practices and the needs of these older adults. Furthermore, the methods and value of the used citizen science approach were evaluated.

## 2. Methods

### 2.1. Design

To assess the specific value of Anne4Care for the envisaged user group, a wide variety of aspects of living with dementia and having a migration background must be reflected in the design and execution of the study. This was achieved by the intensive involvement of older adults and their carers from the early design of the study to analyzing the results and presenting the conclusion. Hence, persons with a migration background in an early stage of dementia participated as co-researchers. There were several stages in this study where researchers and co-researchers collaborated intensively. First, one care professional was consulted regularly during the design of the research. Her contribution was essential to making the final design of this research and understanding how we can collaborate with older adults with a migration background. For example, how much time they could contribute to the research process and how. Second, the topic list that was used in the semi-structured interviews was evaluated and updated by the co-researchers. The co-researchers had a final word considering the formulation of certain questions and interesting topics. Third, the coding matrix used to analyze the interviews was evaluated and updated by one co-researcher. This co-researcher made the matrix complete and specific to their experiences and daily life. Fourth, the conclusion was drawn and presented in close collaboration with co-researchers and researchers. During one presentation, the care professional, a researcher, and a co-researcher presented the findings together to a general audience.

These former steps needed to meet certain conditions. These conditions were drawn from the experience that citizen science has a strong social aspect in which values such as respect and equality play an important role. For example, a good relationship is paramount for collaboration and both co-researchers’ and researchers’ input and adaptations are mutually respected. It also means that care professionals monitor the health status of co-researchers and put research actions on hold if necessary. Finally, having an eye for reciprocity as giving credits but also in the sense that collaboration can have both indirect (e.g., better technology as the outcome of the study) and direct health benefits since being a co-researcher brings new social contacts, new mental challenges, and purposes into their lives.

### 2.2. Setting

This research was part of the TOPFIT Citizenlab program, a research and innovation program in which citizens, healthcare professionals, and companies join forces with researchers to develop and implement technology for health. Furthermore, this program investigates the applicability, results, and contribution of citizen science methodologies in different settings. The present study involved clients with a migration background who visited the activity program of a healthcare organization. Two organizations (IMEAN and Alifa) were involved, both situated in the Twente region of the Netherlands. These organizations provide care to clients with a migration background with cognitive impairments. IMEAN solely provides care to male older adults and Alifa provides care to both male and female older adults. Most clients visit these care organizations two times a week to participate in activities. Three care professionals participated in this study, who all took care of the clients and spoke their native language. This was important to create a safe and trustful environment for the clients and give them a voice when they were troubled with conversating in the Dutch language. The Anne4Care device was installed in the homes of the clients/co-researchers. None of them were familiar with or made use of comparable technology. The team of researchers closely collaborated with the care professionals and Anne4Care company during the design phase. Decisions regarding the procedure of recruitment, obtaining informed consent, data collection, and analysis were made jointly. The recruitment was performed by the care professionals.

Before recruitment and data collection, ethical approval was obtained from the University of Applied Sciences Saxion Ethical Advice Committee (reference number SEAC-2020-005). The co-researchers were personally informed about the study before the start of the test period with Anne4Care. They gave written consent and were informed about their right to withdraw at any moment. Data were anonymized, confidentiality was maintained, and the data will be retained for 10 years.

### 2.3. Stakeholders

There are three main stakeholder groups within this project: A core team consisting of five researchers, a project manager of Citizenlab, and a developer of Anne4Care; a group of three care professionals involved in two different organizations; and a group of 13 clients with a migration background. The core team had biweekly meetings in which one of the care professionals was always present, and the care professionals were all involved during the interviews. All clients were involved as co-researchers, and as compensation for their participation in this research, they could use the Anne4Care technology for free. The co-researchers had an established care relationship with the care professionals and participated jointly in organized group meetings about Anne4Care. In addition, the co-researchers were in contact with the core team during interviews. The older adults did not receive any training in research activities to become co-researchers. In collaboration with the care professional and the older adults, we discussed who would be capable and who felt comfortable performing which task. All co-researchers received training and assistance to use the Anne4Care technology. The basics of the technology were explained by their care professionals and they could reach out to a help desk for questions.

### 2.4. Informed consent procedure

The informed consent procedure was an important activity for establishing relationships and trust between the researchers and co-researchers. The language barrier and level of literacy needed to be taken into consideration with older adults. During an introductory meeting, the researchers visited IMEAN to meet two care professionals and five clients. The professionals working with the clients had a key role as mediators. They already knew their clients very well and speak their language. They were constantly monitoring the situation and translating the informed consent statements into Turkish. They also translated the questions of the clients to the researchers. The informed consent form was explained, stressing that there was no pressure to participate and that they could decide to leave the study at any moment. We found this meeting extremely important because it laid the foundations of a relationship with the clients who became our co-researchers, while at the same time stressing the importance of citizen science and their role as co-researcher.

After evaluating this first meeting with the care professionals, they suggested working on a video explaining the study and the steps of informed consent. This tool supported care professionals in explaining the project to new clients. A video was created in the Turkish language. It was shown during two informative evenings at Alifa and was received well by the clients. It helped evoke questions from clients and address the concerns of some of the participants. Working closely with the care professionals meant that we regularly spoke with them regarding the atmosphere and feelings in the client group. This strengthened our relationship with the clients, as we could address the topics of interest during the interviews. To provide a few examples, there were fears about Anne “listening in” to conversations at home or worries about the cost of electricity. Other considerations of the clients included the follow-up when the study would end. Will they be allowed to keep Anne4Care? These issues were uncovered during the first meetings and were further discussed during conversations between the researchers and co-researchers.

### 2.5. Data collection

Since Anne4Care was introduced as a response to the COVID-19 pandemic, the data collection took place during the pandemic. Data were collected between September 2020 and November 2021. The semi-structured in-depth interviews took place at the care organization or at the home of the co-researchers. The location was chosen according to the preference of the co-researcher. During the interviews, a care professional was always present. In some cases, they assisted in the role of interpreter. Even though the researchers did not understand the co-researcher when they talked in the Turkish language, we experienced that the co-researcher made contact with and talked toward the researcher who asked the question. Co-researchers were invited for two interviews. The first interview was planned shortly after the introduction of Anne4Care in their home, and a second interview 4 months later. Five researchers conducted the interviews. The follow-up interviews were conducted by the same researcher(s) (Table 1).

**Table 1:** Three steps of data collection, method, number of included co-researchers, and purpose of each step.

Data collection	Method	Co-researchers	Purpose
First interviews	Semi-structured interview	13 co-researchers	1. Understand the lives and care needs of the co-researchers 2. Explore their expectations regarding Anne4Care 3. Discuss previous experiences with care and technologies
Second interviews	Semi-structured interview	8 co-researchers	1. Explore the experiences with and suggested adjustments of Anne4Care 2. Discuss their experiences with the current and ideas for future roles as a co-researcher
Interviews with care professionals	Semi-structured interview	4 care professionals	1. Explore their role and experiences as a professional in the use and implementation of Anne4Care 2. Discuss their experiences in working together with the co-researchers

A total of 13 first interviews were conducted with all involved co-researchers. The interviews lasted between 30 and 60 min. The topic guide was developed in collaboration with one co-researcher and the care professionals. The first interview aimed to start the collaboration between researchers and co-researchers and get further acquainted with each other. The discussed topics included (1) the lives and care needs of the co-researchers, (2) their expectations regarding Anne4Care, and (3) previous experiences with care and technologies (Supplementary material 1).

A total of 8 s interviews were conducted with the same co-researchers who participated in the first interviews. Five of the co-researchers were not interviewed two times by the researchers due to COVID-19 illness. These interviews lasted between 20 and 45 min. The care professionals had a conversation with the five missing co-researchers, and the outcome of these conversations was shared with the researchers. The topics discussed during the second interviews included (1) the experiences with and suggested adjustments of Anne4Care and (2) their experience with the current and ideas for future roles as a co-researcher (Supplementary material 1).

Before, during, and after the interviews, the care professionals regularly organized meetings with all co-researchers. The co-researchers were asked to bring their Anne4Care. During the meetings, they shared experiences and were able to help each other with difficulties. The care professionals were present to help as well. Afterward, they shared their experiences with the core team.

Four semi-structured interviews were conducted with the care professionals. These interviews took place at the care organization and lasted for 60 min. These interviews aimed to talk about their role and experiences as professionals in the use and implementation of Anne4Care and working together with the co-researchers (Supplementary material 2).

### 2.6. Data analysis

All interviews were audio recorded and transcribed verbatim. The transcripts were made in English and Dutch. All Turkish spoken words were translated by an interpreter during the interviews. Due to our close collaboration with the care professionals, we have good reasons to assume that the translations were sufficiently accurate. Only the translations were part of the transcripts. Content analysis was performed on all transcripts. Inductive coding was applied to observe and combine various aspects into overarching themes (32). During the first step, open coding was used to identify relevant themes. The concepts of migration background, technology for care, and culturally sensitive care were taken in relation to the themes determined with the inductive coding process. Three researchers performed the analysis of two transcripts and compared the coding. A coding matrix was developed comprising these themes. Thereafter, one transcript was anonymized and coded together with a co-researcher. The coding matrix of the researchers was presented, and the co-researcher suggested many additional themes, such as unawareness of the older adult regarding the risks when the technology would fail, dependency on technology or care in general, and disappointment when the technology did not answer to expectations. This led to the final coding matrix, which was used by one researcher to continue the data coding (32). The co-researcher was only involved in coding one transcript due to the cognitive load and concentration that the analysis asks of the co-researcher. The researchers discussed the coding at biweekly meetings. Translation of quotes from the transcripts to English took place in preparation for the findings. Software package NVivo11 was used for the data coding.

## 3. Findings

First, we present an overview of the co-researchers and a case description of one co-researcher. Then, the findings are presented and structured along the materials, competencies, and meanings involved in the use of Anne4Care by our co-researchers. Furthermore, we highlight how the practices of others in the network of the co-researchers were affected. Thereafter, the implications of being a co-researcher are further explored.

### 3.1. Co-researchers

A total of 13 co-researchers collaborated in this project. Since IMEAN solely provides care to male older adults, we recruited a higher number of male co-researchers; 10 of the 13 co-researchers were men, and their ages varied from 52 to 83 years. One had British nationality, but all others had Turkish nationality (Table 2). A case description of one co-researcher is presented in Box 2 to show an example of the living context of the co-researchers who were involved in this research.

**Table 2:** Demographic characteristics of co-researchers (*N* = 13).

Co-researcher	Gender	Age	Nationality
1	Man	65 years	Turkish
2	Man	82 years	Turkish
3	Man	83 years	Turkish
4	Man	65 years	British
5	Woman	71 years	Turkish
6	Man	80 years	Turkish
7	Man	52 years	Turkish
8	Man	59 years	Turkish
9	Man	77 years	Turkish
10	Woman	65 years	Turkish
11	Man	73 years	Turkish
12	Man	83 years	Turkish
13	Woman	70 years	Turkish

**BOX 2 Case description of co-researcher EE.**

Mrs. EE is 70  years old and born in Turkey. Thirty years ago, she arrived in the Netherlands together with her husband, and they live in the Eastern part of the country. Mrs. EE does not have many friends in the neighborhood and does not have much contact with neighbors. This problem increased due to the COVID-19 pandemic because it was more difficult to visit each other, prepare dinner together, or visit the mosque. There are no relatives in the Netherlands to assist with care or daily activities. Mrs. EE has heart failure and diabetes, and deteriorating auditive and visual capacities. Furthermore, she has an early stage of dementia and visits daycare for 2 days a week. On the other days, she is at home where she does some housekeeping tasks and uses a scoot mobile for outdoor activities, such as grocery shopping.

Anne4Care was introduced in the home of both Mrs. EE and her husband. They each received their own tablet to use Anne4Care. Mrs. EE was already using different technologies such as a mobile phone and laptop, especially to search for information on the Internet or to play games. Anne4Care was introduced at first only to be used for reminders of medication and appointments. She did not use the video conference possibility. After a couple of months, the functionalities of games and listening to the radio were added. These additional functionalities were welcomed and used often. Furthermore, the reminder option was valuable for Mrs. EE because she uses many different medications, and alerts were set to remind her of prayer times. Finally, reminders were introduced for the preparation of dinners including recipes and to turn off the gas after preparing dinner.

### 3.2. Technology in the lives of older adults

To understand the role of technology in the lives of older adults, the interviews focused on the perspective of the co-researchers on technology, the use of technology in general, and more specifically the experiences with Anne4Care. In this section, we inquired into how technology could become part of the daily lives and particular practices of the co-researchers. Following the social practice approach, in our analysis of the findings, we paid particular attention to how meanings, material elements, and competencies influenced and featured in the ways Anne4Care was approached by the older adults, how they used it, and the needs they voiced. Furthermore, we considered not only individual practices but also how they affected relations with others, in particular family members and carers.

#### 3.2.1. Meanings and expectations

Most co-researchers preferred to use Anne4Care throughout the day as it gave them a purpose and was valuable to assist with health-related tasks. Anne became a part of their home and routine. In the morning, Anne started their day by saying “good morning.” This was a warm welcome and gave the feeling there was someone else at home. They also felt comfortable with the avatar of Anne4Care being present in their home. Although the avatar was a blond woman, it was not considered important to change toward, for example, a Turkish woman. Also, the name had a positive cultural meaning according to the co-researchers with a Turkish background, because the word “Anne” means mother in the Turkish language.

Most co-researchers were benevolent to work with Anne4Care, welcomed technology in their life, and preferred to have virtual assistance at home. They were interested in Anne4Care because it is a device communicating with them in their native language. The co-researchers stated to use technology as it makes their lives easier, for example, by allowing them to communicate with family and friends in their home country, helping them to cope with memory loss, or supporting them with the Dutch language. Furthermore, technology should provide health benefits. The co-researchers considered this as particularly important for them, as their family often lives in a different country and could not support them with health problems or health tasks in their daily lives. Some of these tasks in which Anne4Care could assist is the provision of reminders for medication. However, it could also be used for different, culturally important practices that are relevant for structuring their day, such as reminders for daily prayers.

*“Our older generation thinks about certain things differently than the younger generation. They have different interests to use technology. People from my age, we are interested in the health benefits and how we are supported to reach these.”*—KK.

Half of the co-researchers thought Anne4Care was an entertainment or learning device. There were already some puzzles and games, but the co-researchers pointed out that more advanced puzzles or games are needed to provide more challenges. These challenges are discussed as valuable to cope with dementia and possibly decelerate mental deterioration. Furthermore, language games were discussed as an addition to facilitating knowledge of the Dutch language. The functionalities such as games and puzzles as well as reading a newspaper, listening to the radio, and even managing the agenda were meaningful activities.

*“It is so much fun to work with Anne. For example, when I have a new appointment, I would like to know how to add this to the agenda. Anne keeps me occupied, active, and at the same time more independent.”*—MM.

In addition to the positive ideas about technology, there are some concerns based on privacy issues. Some co-researchers disclosed that they just do not like the internet. They have no idea what could be possible with these modern technologies and who could have access. Some mentioned that people from their generation are a bit slow in accepting something new and have a negative attitude toward technological development in general.

*“They turned Anne off during the nights. I asked why they switched it off and he told me that they were afraid someone could watch through the device. Sometimes we just want to go on too fast, but the technology frightens them, and they must get used to it.”*—Professional A.

#### 3.2.2. Materials and infrastructure

Most co-researchers paid little attention to the multiplicity of technologies they already used. As an exception, one co-researcher was interested in technology during his entire life. He had a collection of older and newer photo and video devices, which assisted him in staying connected with Turkey. A small number had technologies including alarm devices, a flashing doorbell, and a robot vacuum cleaner. The couple who possessed the robot vacuum initially did not expect to use the robot but were eventually satisfied with the result and the easiness of use. Although most co-researchers did not feel very knowledgeable about technology, they had expectations of technology in general as well as of Anne4Care specifically. Considering Anne4Care, most expected to take the device outside their homes also. However, Anne4Care stopped working when plugged into the electricity socket or when it lost an internet connection. This implied that the device could be used at home but not outside, and, for example, medication reminders were not provided when leaving the house. The co-researchers argued that this made the device more useful for housebound people. To support their daily and health practices under varying circumstances, it should be possible to take the device outside.

*“It would be good if they introduce a mobile app that interrogates the same personal details which I could use when I’m out, and then they can remind me to take my medicines when I’m not at home. Now it would only really work for somebody who’s housebound.”—DD.*

In the case of older adults with a migration background, financial resources are often minimal, which makes it more difficult to purchase the technology. Furthermore, the co-researchers had no idea where others could buy such a device and in which stores it could be purchased, and they wondered about the costs when the project would end. Regarding finances, the professionals compared it to the transition from a landline phone to an internet-based smartphone. This was established due to the possibility to call their family and friend in their country of birth free of charge. Thus, Anne4Care would be more likely to be taken up, if it could replace their smartphone and should not include additional costs. Some co-researchers were concerned about the electricity bills because the device needs to be plugged in all day.

*“The idea to spend money or save money is deeply embedded in these older adults. Saving as much as possible is especially important because they need it for tickets to visit family and friends. Therefore, purchasing innovative technologies, paying the electricity bills,* etc. *is a huge problem for them.”*—professional A.

Another suggestion, connected to finances, was made by the care professionals. To improve the care they could provide, there should be an application in Anne4Care to assist with financial and administrative tasks. This kind of assistance would add more value to the device for some of the co-researchers because it gives them a deeper understanding of their financial situation and possibly more independence.

#### 3.2.3. Competences, learning, and support

The professionals argued that the introduction of Anne4Care in the homes of older adults needs time, attention, and understanding of their situation and cultural background. The care professionals strive toward a smooth introduction of technologies such as Anne4Care to improve independent living. In the future, introducing the technology in an early phase seemed necessary due to the beginning of dementia and the time the appropriation process takes. According to the professionals, this would be possible if they introduce it to each of their new clients. However, it may also be that, in an earlier phase of dementia, the clients could have more difficulties seeing the value of the device. The embedding of technology introduces new practices of support in addition to the current care practices. For example, additional help could be provided by the care organization, but assistance should also be provided at home. The questions of how this support is arranged and who will have which task need to be determined.

*“At the start of the project, the coordination was done by someone else and I was not involved in the decision to start using this technology. The moment we started to introduce Anne to the clients in our organisation, that was the moment I got involved. I consider this as a well-aligned process. Then the introduction at the homes of our clients went through different phases, and with some it took more time than with others. The phases for example included setting-up the device, get started, explanation of different functions, and testing the device together. At every home visit, we need to send some time with the device to make it part of our clients daily practice and our care practice.”*—Professional CC.

According to the care professionals, the involvement of a care professional they trust was critical to introduce technology into the lives of older adults with a migration background. Initially, most co-researchers had some hesitation and the care professionals as well as other co-researcher who were already involved in the project had to convince them to use Anne4Care. However, once they got familiar with the device and embedded it into their daily routines, they did not want to miss it anymore, as it gave them purpose in their daily activities and helped with staying more independent in health tasks.

All co-researchers were eager to participate and use Anne4Care. However, a remark made by most of them was that they needed assistance to use technology in general. Many needed help to use their smartphone, for example, to send a message or translate a Dutch message. They lacked the appropriate knowledge and familiarity with digital technologies. They came to the Netherlands to work in sectors where technologies did not play an important role. Thus, they did not come across many new or digital technologies during their lives. To use technology such as Anne4Care, they need to learn new skills. Although learning is acknowledged as valuable for coping with dementia, learning something new is challenging, especially due to their deteriorating memory and obliviousness.

*“We do not understand much about technology! I cannot handle it well. When we would have had the ability or need to use technology in an earlier stage that would have been easier. There is so much under development and now, storing new knowledge is a slow process.”*—CC.

Although they recognize their suffering from obliviousness and necessary time to learn something new, some wanted to learn how Anne4Care operates. This provided distraction during the day and challenged them while discovering more about technology.

*“These are all new things for me which I would like to use, but I need to know and learn how to do it, or I will not be able to use it. That would be such a pity.”*—KK.

One co-researcher suggested the possibility to develop a short course on technology to help with the learning difficulties. He mentioned that repetition is an important aspect of such a course, all the more for older adults with dementia. Repetition is necessary for people with dementia to provide a more permanent understanding.

*“They have explained this once or twice, but then you need to remember. I need someone to practice the new things in order to actively remember.”*—LL.

In addition to repetition, the language barrier should be considered. For the co-researchers, it was important to collaborate with people who can overcome the language barrier and are familiar with low digital literacy. This collaboration should become part of the current care for older adults. Next to a course, the co-researchers emphasized the usefulness of the joint meetings at the care organization, which allowed them to share knowledge and help each other to work with Anne4Care. These meetings were experienced as fun and worthwhile. It gave the co-researchers the possibility to ask questions and use the technology. This option of collaboration can be integrated into current care. Furthermore, the co-researchers argued that it is important to ensure that they can communicate in their native language, all the more for older adults with dementia, as second language skills may suffer. The addition of an employee at the Anne4Care service desk who spoke their language was noted as meaningful by all co-researchers. If they came across difficulties in using the device, they could reach out to this employer and receive immediate help without the interference of an interpreter.

### 3.3. Family and care relationships

The introduction of a technology such as Anne4Care in the lives and homes of people implied that people in their surroundings are faced with it as well. One co-researcher disclosed that he had regular discussions with his partner before Anne4Care was introduced. His partner had the task to remind him to take medication, and then, there was a device at which he did not become angry. This improved their relationship and lowered the care burden of the partner. In addition, all care professionals acknowledge that the tasks of informal caregivers are lowered, especially due to the reminders provided by the device. However, another partner had more trouble with Anne4Care because she had to do additional tasks to make the device work rather than being relieved from them. The partner not only had to learn how to use Anne4Care but also keep the device up-to-date, which took a lot of time.

*“We spent probably a whole Thursday afternoon trying to figure out how to make it work. Also, now my partner must put the medicines in manually and type all these difficult medical words. Oh, a lots of medical words, brand names, she has to add all and the frequency per day and what time of day it needs to be taken. And do that all manually does not take work away from her, but it puts work on top. Also, to put my appointments from the diary, I have a paper diary, and she must go through and copy all of these and put them in.”—DD.*

Another discussion with the professionals concerned the possibility to provide more personalized care. More personalized care could improve their relationship with the clients. Anne4Care provides the opportunity to adjust care to different disabilities and help with personal needs, such as reminders for daily prayers or turning off the gas after cooking activities. The care professionals knew about the lower ability to remember to turn off the gas of one co-researcher. To stay at home, the first advice was to stop cooking meals, which was not the most healthy solution for this co-researcher due to diabetes. With the possibility to add all kinds of reminders in the device, the co-researcher could live longer in her home and make her own healthy meals at home by receiving recipes, shopping lists, and reminders to cook and turn off the gas. In addition, in the example below, the care professional came across the difficulties her client had with household chores due to reduced arm strength.

*“With the use of Anne at the home, we get more insight into the lives of our clients. For example, this man seemed to function very well, but with the use of Anne we encountered a disability with which he could have some assistance from us. You see how we can also ignore some care needs.”*—Professional B.

### 3.4. Being a co-researcher

After the extensive informed consent meetings, some clients were more open and directly willing to participate while others were more reluctant. They mutually talked about the technology and the research and some convinced others to participate. In the end, all clients present during the meeting became our co-researchers. The name of the co-researcher gave them a feeling of pride and being part of a development process. They all knew that Anne4Care was still in a developmental phase and did not expect that everything was working optimally.

*“I know Anne still needs to be improved, it is in the development phase. Yes, there are some things that are not working as they should, but that is why I am involved. I will test it at home to face all ailments and provide important feedback to adjust Anne where needed.”*—HH.

Most mentioned the group meetings during which they shared their experiences and knowledge and learned from each other.

*“We share the needed adjustments for the device, but also what we expect will be discussed. Everyone is free to share personal needs and everything can be brought onto the table. Where one of us would like to have a different interface for the medication, someone else needs an alarm button.”*—BB.

These group meetings made everyone feel valuable and being part of the research project. Giving their opinion made them feel more important and of influence on the final development. For older adults, this social component in the project was particularly important. Practicing together increased their motivation to learn and use the technology. One of the co-researchers also compared the home situation where her husband had to learn to use Anne4Care on his own or together with his wife with the group meetings where he is with other men. She claims that he is more motivated when he is around other men.

All co-researchers want to recommend Anne4Care to others, and a small group would even be part of promoting activities. They have ideas concerning where to find older adults with a migration background and how to engage them in meetings about modern technologies. However, they mentioned some barriers such as fewer social connections due to COVID-19, low digital literacy, and unknown availability and costs of the technology.

*“I would like to recommend this to others, share my knowledge in a group. However, I expect most of them to say ‘I cannot even write or read’… Yes, most of them are analphabetic and do not have had any education. Others would be hesitating because they expect a lot of challenges while learning this innovative technology, they would claim to have too little brain capacity. Still, I would very much like to recommend it.”*—EE.

## 4. Discussion

This study aimed to explore the role of digital technology in coping with dementia in the lives of older adults with a migration background and to explore possibilities to engage and collaborate with older adults, as our co-researchers. Considering the first research question, digital technologies for care may help older people with dementia to stay independent and connect to loved ones in their country of birth. The technology should fit into their lives, contain adjustable language settings, and can be used both inside and outside their homes. Important while collaborating with co-researchers, regarding the second research question, is acknowledging their expertise and needs and working together in short iterations to adapt the technology to their specific needs and situations. The group sessions in which the co-researchers shared findings and worked together with others with a similar background and the same language were experienced as very valuable. The following sections will discuss four topics in more detail, all revolving around the relations between the four core “entities” in our study: co-researchers, researchers, care professionals, and technology: (1) how to reach older adults with a migration background to experiment with the use of digital technology, (2) how to build a relationship with them to assist them in using technology and to collaborate with them in research, (3) the value of the engagement and collaboration across the research process and the need to adjust the engagement preferences and capabilities of the co-researchers, and (4) the role of technology in their lives. The discussion ends with the strengths and limitations of this research, an overall conclusion highlighting the main insight, and recommendations for practice and future research.

### 4.1. Reaching older adults with a migration background

Based on our research, we identified several challenges regarding the development and implementation of digital technologies for coping with dementia for older adults with a migration background. Regarding our research questions, there are several challenges we will further discuss. One of these challenges in healthcare and social work is to reach people with a migration background. In our conversations with older adults, it became clear that, for making them aware of care technologies, providing culturally sensitive care, or collaborating with them in the development or implementation of the technologies, it is necessary to reach these older adults directly. However, they also stressed that most older adults with a migration background do not want to use digital technology or make use of a health service. Health services often have difficulties reaching people with a migration background, also because little culturally sensitive care is provided and understood (4). Some of our co-researchers had negative experiences with previous healthcare organizations and preferred specialized care organizations such as those involved in our study.

To reach older adults with a migration background to experiment with technology, different suggestions were given by the co-researchers. The most important suggestions were the support of the older adults among each other or support through specialized care organizations. The involved care professionals and researchers experienced that reaching older adults willing to experiment with the technologies was challenging. Some studies described that people with a higher social status are more likely to be early adopters of new technologies and people with a lower social position adopt technology later (33). Most older adults with whom we collaborated did not identify themselves as early adopters of technology and would not have experimented with the technology if the care professional did not approach them.

### 4.2. Building a relationship

In addition to involving a specialized, trustworthy organization, as a second element, our findings showed the importance of the relationship between older adults with a migration background and their care professionals. This was important for getting interested in and accepting the new technology for care into their lives. Due to unfamiliarity with technology, a relationship of trust with a care professional was crucial in combination with culturally sensitive care. The position of the care professional was important to convince older adults to use technology. She understood culturally sensitive care, had the same nationality, spoke their language, and had a relationship of trust with the co-researchers. In the early stage of this study, the researchers and care professionals had extensive discussions with older adults. These discussions revealed barriers and worries for this specific group of older adults, such as anxiety, language, the added value of technology, and cost of electricity use, and helped to create a climate of trust.

In addition to the relationship with the care professional, the same applies to the relationship with the researcher. Researchers must take time to introduce themselves, build trust, and work together with the co-researchers. Acknowledging and valuing the expertise of co-researchers gave them a feeling of pride. Other studies also showed that trust in researchers has a significant role in enhancing cooperation with co-researchers (34). Incorporating the views and needs of vulnerable populations is the first step (34), and an active role as a co-researcher is the next.

### 4.3. Collaboration with the co-researchers

Third, the continuous, collaborative involvement of the co-researchers across different steps of the research proved very valuable both for the research process as well as the adoption and integration of the technology into user practices. It appeared, however, also important to allow for some flexibility in how individual co-researchers were involved. The collaboration with co-researchers revealed various needs and desires toward future developments of Anne4Care. The co-researchers in our study made a lot of suggestions to improve the technologies to make them fit in their lives, such as having an alarm button. The problems they experienced were necessary to improve the device and make it more useful. Another device, “skype on wheels” (19), had a low engagement among older adults after implementation in the homes due to multiple barriers, which could have been overcome with a collaborative process (19).

The question of how to engage and collaborate was also discussed with the older adults during the interviews. One suggestion was to collaborate in reaching out to other older adults with a migration background. In this study, they received the role of a co-researcher and had the option to support others in getting to know about the technologies for care or even assist in using them. They have the knowledge of where to find the target population and how to approach or talk about digital technology. While a citizen science approach strives for a high level of engagement, we allowed the co-researchers to engage in different degrees and divide tasks, depending on what individuals felt desirable and feasible for themselves. Most of the co-researchers felt capable of at least testing the device and collaborating in the workshops and interviews to discuss their experiences and needs for adjustments to the device. Only a small group was involved in discussing the topics for the interview guide and one co-researcher was involved in analyzing the data. While it would have been valuable to include more co-researchers during the analysis, it also became clear that research interests needed to be balanced against the risk of overburdening the co-researchers.

### 4.4. Technology in the lives of older adults

The last point is connected to our first research question and elaborated on how to relate to technology for care and embed it in the lives of older adults. According to the co-researchers, it was rather easy to fit the technology into their home and home routine; this was also observed by the researchers and care professionals involved in this study. The fact that it was a tablet, a physical object, made it an artifact with which people started to talk in their native language. This affectionate reaction is achieved with such artifacts, rather than with apps (35). Apparently, including an avatar that invoked culturally positive meanings facilitated the creation of such an affectionate relationship as well. When a new artifact is implemented, it co-shapes daily practices, the actions people take, and the relationship among the user, the artifact, and the surrounding (36–38). The older adults experienced a connection with the technologies brought into their homes, and they even started to trust the technology. On the one hand, they interacted, talked, and reacted to the technology, and on the other hand, they felt a connection and the artifact became an element in their routines and practices. However, before this connection is possible, older adults need to open themselves to using technologies for care.

As Kouvonen et al. (18) argue, older adults with a migration background and ill health experience more barriers to using technology than those with better health. They argued that the technology should be adaptable to those with specific cultural needs to facilitate adoption. The care professionals in our study also argued that it is important to invest time in providing support, especially with the introduction of technology. They invested a lot of time to explain the technology and collaborate with the older adults when they used the technology for the first time. Older adults need to perceive that they can and why they should use digital technologies for care. For this, it appears necessary to assess life and health-related differences to identify which particular technology older adults could and would use and which might differ between native and migrant older adults (39–41). Similar to the division of tasks during the experimentation of digital technologies, also the eventual use of these technologies will differ from one to another. Mitchell et al. (16) also underlined that the use of specific applications is not similar across older adults with different nationalities, which could even cause health disparities.

### 4.5. Strengths and limitations

The involvement of older adults with a migration background in an early stage of dementia as co-researchers is considered a strength of this study. However, those with a moderate stage of dementia may have different experiences. Our collaboration strategy allows matching the ideas and needs of the co-researchers with the technology under development (42). In addition, the researchers worked closely together with care professionals to get an in-depth and multi-stakeholder perspective on the development and (first) use of technology. The involvement of the care professionals was also clear in the group meetings they organized at the organizations. Although an interview lasts only for a short time, being part of a research project is the start of sharing stories and building new collaborations (43). This also was the outcome of the collaboration between co-researchers and researchers within the involved care organizations. Close collaboration with care professionals was also of paramount importance since we were dealing with a group of easily overburdened people.

When interpreting the results, the following points must be considered. First, most co-researchers were men with a Turkish background. In addition to that, female co-researchers appeared to be more open toward technology, and the analysis did not show major differences between their male counterparts and the small number of female co-researchers. It is unknown whether the findings are also applicable to older adults with different migration backgrounds. In this study, the device Anne4Care was introduced in the two care organizations IMEAN and Alifa. In these organizations, there were mainly male older adults with a Turkish background, and it was therefore not possible to include co-researchers with a different migration background. Further research should also include care organizations for female older adults. Second, the device Anne4Care could have influenced and directed the findings due to experiences with this specific device. However, the device gave grips to the understanding of technology, and as O’Reilly-de Brún et al. (33) also described, implementing the device enabled the researchers to reach these older adults and enhance a meaningful contribution.

## 5. Conclusion

Technologies to cope with dementia can become part of the care practices and lives of older adults with a migration background. However, older adults need assistance from care professionals to understand the added value of technology and learn how to use it. The co-researchers need additional competencies to work with digital technologies, but the learning process to use technologies is also considered purposeful in their daily life. Furthermore, not all co-researchers had the materials, such as an internet connection or financial resources, to use the device, and the current care practice needs to adapt to the technology. Technology assists with independency, participation, and activities in daily life. To keep using technology, it should provide health benefits, have the option to use it inside and outside the home, fit in their care and lives, and include language settings.

The involvement of the older adults with dementia as co-researchers made them feel valuable and equal partners during the research project. Involving older adults in the development of technology, acknowledging their expertise and needs, and working together in short iterations to adapt the technology to their specific needs and situations were experienced as valuable by the researchers, older adults, and care professionals. In addition, it became clear that the formation of relations between the involved groups, as well as the technology, was an important element in the process.

Probable goals for future research could include a similar study with more co-researchers as well as co-researchers with different demographics, such as a moderate state of dementia, more female older adults, and different migration backgrounds. This could be reached through the investigation of technologies for care in more and different care organizations. Furthermore, this research showed needed changes in care practices. A recommendation for care organizations is to be aware of new developments in technologies, be aware of the need and possibility to provide culturally sensitive care with these technologies, and consider the needed pathway of introducing and support in using the technology. This study provided some first insight into these pathways, but more in-depth understanding and research to design strategies are required.

During the execution of this research, we took a citizen science approach to investigate the engagement and collaboration of older adults with a migration background. At the same time, we focussed on the social practices of these older adults with technology. In this research and during conversations with the co-researchers, we have obtained knowledge and were able to discuss some important elements in the engagement and collaboration, but a more in-depth understanding would provide additional answers to our second research question. Further understanding could be obtained, for example, by varying the research process.

## Data availability statement

The raw data supporting the conclusions of this article will be made available by the authors, without undue reservation.

## Ethics statement

The studies involving human participants were reviewed and approved by the University of Applied Sciences Saxion Ethical Advice Committee (reference number SEAC-2020-005). The patients/participants provided their written informed consent to participate in this study.

## Author contributions

CL, ES, ZM, and MB conducted the interviews, read, and compared the findings. At biweekly meetings between the project team and a care professional, peer debriefing took place. ES had a close collaboration with the co-researcher during the analysis. All authors contributed to the design, preparation of the study, writing the manuscript and approved the latest version of the manuscript.

## Funding

This study was powered by the Twente Regional Deal and received financial support from the Central Government’s Regional Budget, the Province of Overijssel, the Region of Twente, and the Twente Board. The funders had no role in study design, data collection and analysis, the decision to publish, or preparation of the manuscript.

## Conflict of interest

The authors declare that the research was conducted in the absence of any commercial or financial relationships that could be construed as a potential conflict of interest.

## Publisher’s note

All claims expressed in this article are solely those of the authors and do not necessarily represent those of their affiliated organizations, or those of the publisher, the editors and the reviewers. Any product that may be evaluated in this article, or claim that may be made by its manufacturer, is not guaranteed or endorsed by the publisher.

## Abbreviations

Anne4Care: anne for care
COVID-19: coronavirus
IMEAN: consultancy and care organization for older adults with a migration background
PVA: Personal Virtual Assistant
TOPFIT Citizenlab: research and innovation program to use citizen science and improve health and wellbeing of citizens
