# Supplementary material for: Engaging older adults with a migration background to explore the usage of digital technologies in coping with dementia
Source: Front Public Health. 2023 Apr 14;11:1125834. doi: 10.3389/fpubh.2023.1125834 (PMC10140574; doi:10.3389/fpubh.2023.1125834)
Supplement: Supplementary file 1 [file Data_Sheet_1.DOCX]

Engaging older adults with a migration background to explore the usage of welfare technologies in coping with dementia

Catharina M. van Leersum^1*^, Kornelia E. Konrad^1^, Egbert Siebrand^2^, Zohrah B. Malik^3^, Marjolein E. M. den Ouden^3^, Marloes Bults^3^

^1^Science, Technology, and Policy Studies, Faculty of Behavioural, Management, and Social Sciences, University of Twente, Enschede, the Netherlands

^2^Ethics and Technology Research Group, Saxion University of Applied Science, Deventer, the Netherlands

^3^Technology, Health & Care Research Group, Saxion University of Applied Science, Enschede, the Netherlands

*** Correspondence:**Catharina Margaretha van Leersum
c.m.vanleersum@utwente.nl

# Supplementary material 1. Interview guide & follow-up interview guide – older adults with a migration background

This is the English translation of the interview guide that was used both during the first and follow-up interviews. The introductory questions were only used during the first interview. The follow-up questions regarding technology and the question regarding collaboration/participation in the project were only asked during the follow-up interviews. The questions about Anne4Care and questions for informal caregivers were used during the first as well as the follow-up interviews.

*Introduction*

- Can you tell us something about yourself?
  - Age
  - Educational level
  - Nationality
  - Living situation
  - Social network
  - Health status
  - Informal caregivers
- What do you usually do in a day?
  - How does your day look like?
  - What do you like to do in your life?
- What is important to you regarding your personal health?
  - What is your definition of health?
  - How would you rate your own health (on a scale of 1-10)?
  - What could contribute to improving your health; what do you need?
- Do you use technology or digital tools in daily life?
  - What technology (digital tools) do you use at home?
  - And in your life with dementia (think of mobile phones, fall prevention, alarm bell, video bell application etc.)?

*Questions about Anne4Care.*

- When did you receive Anne4Care?
- How do you experience Anne4Care?
  - Pleasant / unpleasant?
  - Helpful / unhelpful?
- How have you used Anne4Care so far?
  - Can you give examples of your use of Anne4Care?
  - Can you show the functions you have used and how?
- Which advantages do you experience with Anne4Care?
- Which disadvantages do you experience with Anne4Care?
- What are improvements for Anne4Care?
- Would you like to continue using Anne4Care?
  - What are your reasons to continue / discontinue using Anne4Care?
- Do you need Anne4Care in your life?
  - Could you elaborate on that?
- What is the added value of Anne4Care (daily structure, maintaining social contacts, performing meaningful day activities)?
- Do you need (or have had) help in using or understanding Anne4Care?
  - If so, from whom?
  - Do you need more support or training in the] use of Anne4Care?
    - If so, from whom?
- Do you expect your health or your daily life to change because of using Anne4Care?
- Do you think Anne4Care will change your relationship/contact with your healthcare professional?
- Do you think that technology, like Anne4Care, improves healthcare?
- Would you recommend Anne4Care to others (i.e., peers)?
  - What are your reasons for recommending / not recommending?

*Specific questions for informal caregivers.*

- Do you expect a changing role as a caregiver due to Anne4Care?
  - What are the reasons to expect a change or not?
- Would you like to help in using and understanding Anne4Care?
  - How do you want to provide help?
  - Would you need help to assist in using Anne4Care?
    - If so, from whom would you like to receive help?

*Questions for the follow-up interview regarding technology.*

- What is your opinion on technology (in general)
  - What can technology bring for you? Do you imagine good things or also anxious things?
- What can technology mean for your health and healthcare?
- Which technologies do you currently use in your life and how often?
  - What is the reason for using these technologies?
- Which technologies would you like to use?
  - Is there anything in your life where technology could be helpful?
  - What should technology deliver?
  - What would you expect from the technology?

*Questions regarding reaching and (long-term) participation/collaboration with target group.*

- We would like to carry out this research in collaboration with you. Do you have ideas how we can do this?
- What was your reason to participate in this project?
  - How did you experience participation?
- How should we collaborate with you and other older adults with a migration background during this or other projects?
- How could we reach more older adults with a migration background?
- Through which communication channels could we reach you and other older adults with a migration background?
  - Which messages should we share?

# Supplementary material 2. Interview guide – care professionals of older adults with a migration background

This is the English translation of the interview guide that was used during the interviews with care professionals. Some questions were also used during the interviews with the older adults if a care professional was present and the question fitted the conversation.

*Introduction*

- Can you tell us something about yourself?
  - Age
  - Educational level
  - Nationality
  - Function
  - Work experience
- How does an average day look like for you in terms of providing care?
  - How often do you have contact with clients and informal care givers?
  - To what extent do you provide remote/care at a distance?
- What is important according to you when it comes to the health and well-being of clients?

*Use of (welfare) technology/digital tools in clients’ daily life and personal environment*

- Which technology/digital tools do clients use at your care organization or at home?
  - For example, mobile phone, fall prevention, alarm bell, video bell application

*Questions about Anne4Care.*

- Can you explain how Anne4Care has been delivered to clients so far?
  - What is striking about this?
  - What could be done better/different?
- How do you experience Anne4Care as a professional?
  - What advantages do you experience with Anne4Care?
  - What disadvantages do you experience with Anne4Care?
- Can you give illustrative examples of how you use Anne4Care?
- How would you improve Anne4Care?
- To what extent do you need Anne4Care? What is the added value for your clients and for you as a professional
  - For example day structure, maintaining social contacts, performing meaningful activities
- Do you need help with use or understanding of Anne4Care?
  - If so, with which aspects do you need help? Do you know by whom to receive help if needed?
- How do you expect the health situation of clients to change as a result of using Anne4Care?
- How do you expect that the care provided to clients will change through the use of Anne4Care?
- Would you recommend Anne4Care to other professionals?

*Questions regarding client–professional interaction*

- How do you prefer to build a relationship with your clients? How do you see the interaction and contact with your clients?
- How do you experience your relationship/interaction/contact with clients and informal care givers who use Anne4Care?
- Has the care you provided changed by using Anne4Care?
  - If so, what kind of change do you experience?
- Has the contact with clients/informal caregivers also changed?
  - If so, what kind of change do you experience?

*Questions regarding technology.*

- What technologies do you currently use and how often as part of care practices?
  - Which technologies would you like to use as part of care practices?
- What do you want to achieve by using technology as part of care practices?
- Do you expect technologies to change your relationship/contact/interaction with clients?
  - If so, how would it change?

*Questions regarding reaching and (long-term) participation/collaboration with target group.*

- How can we reach older adults with a migration background to collaborate in research?
  - Through which communication channels and with which message?
- Do older adults with a migration background have contact with others who have a migration background?
  - If so, in what way and how often?
  - If not, do they want to have more connections with older adults with a migration background?
  - How can older adults with a migration background be involved in reaching the others?
- With which other professionals could we collaborate in this research?
